# Ultrafast carrier thermalization and trapping in silicon-germanium alloy probed by extreme ultraviolet transient absorption spectroscopy

**DOI:** 10.1063/1.4985056

**Published:** 2017-06-06

**Authors:** Michael Zürch, Hung-Tzu Chang, Peter M. Kraus, Scott K. Cushing, Lauren J. Borja, Andrey Gandman, Christopher J. Kaplan, Myoung Hwan Oh, James S. Prell, David Prendergast, Chaitanya D. Pemmaraju, Daniel M. Neumark, Stephen R. Leone

**Affiliations:** 1 Department of Chemistry, University of California, Berkeley, California 94720, USA; 2 Materials Sciences Division, Lawrence Berkeley National Laboratory, Berkeley, California 94720, USA; 3 The Molecular Foundry, Lawrence Berkeley National Laboratory, Berkeley, California 94720, USA; 4 Theory Institute for Materials and Energy Spectroscopies, SLAC National Accelerator Laboratory, Menlo Park, California 94025, USA; 5 Chemical Sciences Division, Lawrence Berkeley National Laboratory, Berkeley, California 94720, USA; 6 Department of Physics, University of California, Berkeley, California 94720, USA

## Abstract

Semiconductor alloys containing silicon and germanium are of growing importance for compact and highly efficient photonic devices due to their favorable properties for direct integration into silicon platforms and wide tunability of optical parameters. Here, we report the simultaneous direct and energy-resolved probing of ultrafast electron and hole dynamics in a silicon-germanium alloy with the stoichiometry Si_0.25_Ge_0.75_ by extreme ultraviolet transient absorption spectroscopy. Probing the photoinduced dynamics of charge carriers at the germanium M_4,5_-edge (∼30 eV) allows the germanium atoms to be used as reporter atoms for carrier dynamics in the alloy. The photoexcitation of electrons across the direct and indirect band gap into conduction band (CB) valleys and their subsequent hot carrier relaxation are observed and compared to pure germanium, where the Ge direct $\left({\Delta E}_{\text{gap},\text{Ge},\text{direct}}=0.8\;\text{eV}\right)$ and Si_0.25_Ge_0.75_ indirect gaps (${\Delta E}_{\text{gap},{\text{Si}}_{0.25}{\text{Ge}}_{0.75},\text{indirect}}=0.95\;\text{eV}$) are comparable in energy. In the alloy, comparable carrier lifetimes are observed for the X, L, and Γ valleys in the conduction band. A midgap feature associated with electrons accumulating in trap states near the CB edge following intraband thermalization is observed in the Si_0.25_Ge_0.75_ alloy. The successful implementation of the reporter atom concept for capturing the dynamics of the electronic bands by site-specific probing in solids opens a route to study carrier dynamics in more complex materials with femtosecond and sub-femtosecond temporal resolution.

## I. INTRODUCTION

Studying carrier dynamics in semiconductors has been an active research field for decades. A profound understanding of carrier scattering and decay mechanisms for both carrier types, electrons and holes, is key to the development of improved photonic devices.^1,2^ Most methods that seek to measure carrier dynamics rely on time-dependent changes in transmission and reflection properties in the optical spectral range by pump-probe spectroscopy.^3,4^ However, optical methods often do not separately resolve the spectral signatures of the electrons and holes directly and simultaneously, which renders capturing a full picture of the carrier dynamics difficult.

**FIG. 1.**
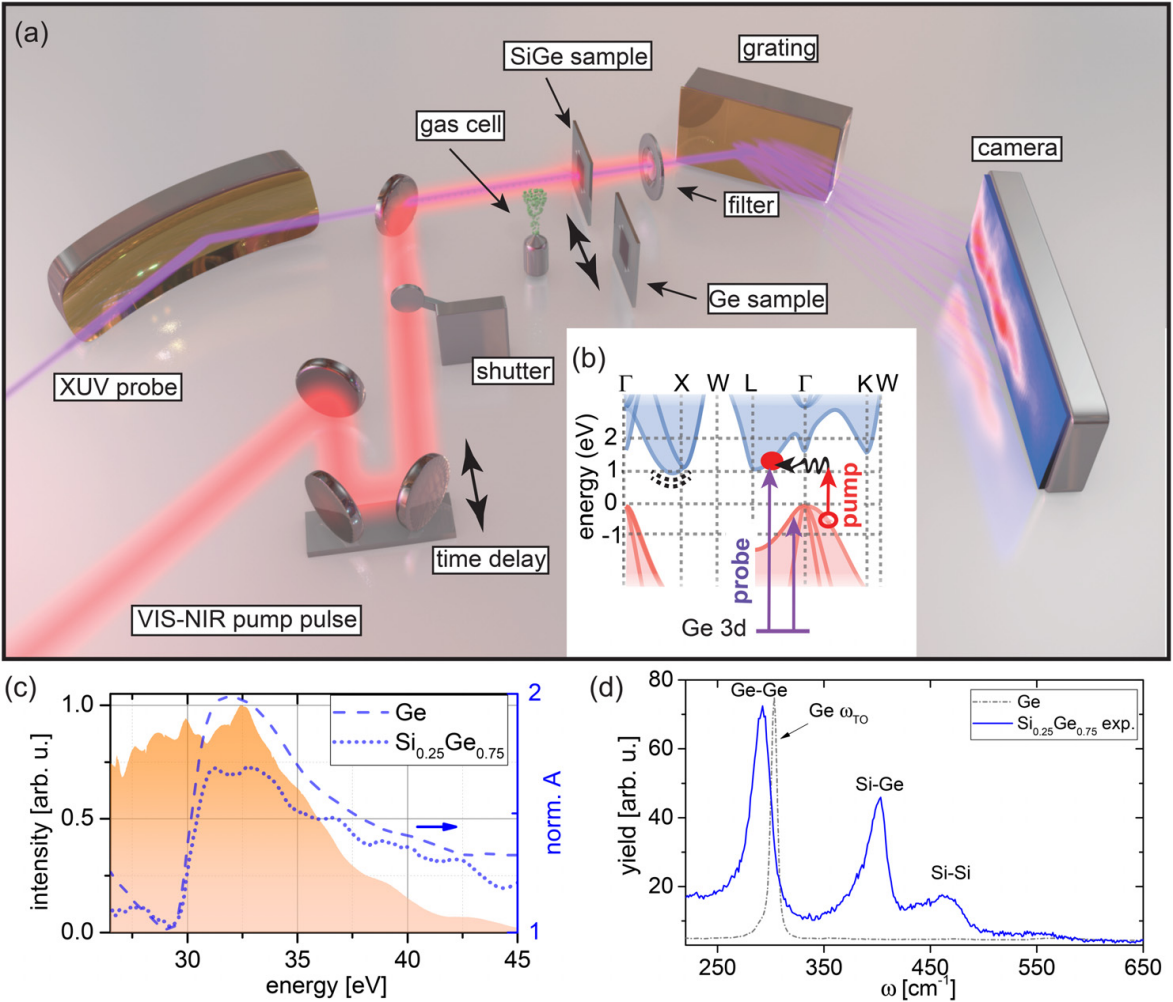
XUV transient absorption spectroscopy on the silicon-germanium alloy. (a) Scheme of the experimental setup. A time-delayed visible-to-near infrared (VIS-NIR) pump pulse and an extreme ultraviolet (XUV) probe pulse are collinearly focused onto the sample. A shutter blocks the pump pulse for acquiring differential absorption spectra that are measured in transmission through a sample with a grating spectrometer. An aluminum filter removes residual pump light before the spectrometer. (b) Band structure and pump-probe scheme in silicon-germanium alloy. A calculated band structure of Si_0.25_Ge_0.75_ is schematically shown [detailed band structure in supplementary material, Fig. S2(a)]. The VIS-NIR pump pulse (red arrow) can excite an electron (filled red circle) assisted by a phonon (black undulating arrow) from the valence (red shaded area) to the conduction band (blue shaded area), here typified into the L valley, leaving a hole behind (open red circle). Likewise, indirect excitation assisted by a phonon is possible into the X and K valleys as well as a direct excitation into the Γ valley by the blue tail of the spectrum (not shown here). The germanium atoms act as reporter atoms for the kinetics with XUV transitions (violet arrows) to both the valence and conduction band from the 3*d* core-levels. The dashed black line beneath the X valley indicates possible locations for trap states in the midgap. (c) M_4,5_ absorption edge of germanium in pure germanium (blue dashed line) and silicon-germanium alloy (blue dotted line) normalized to the pre-edge region. Absorbances were measured relative to an uncoated Si_3_N_4_ membrane of the same thickness. The signal ratio above the edge supports the material fraction of 75% Ge in the alloy. The broadband XUV pulse (shaded orange area) covers the spectral regions associated with the valence and conduction band allowing simultaneous capture of the carrier and band shift kinetics. (d) Raman spectra of the samples used in this work. The monatomic germanium sample shows a strong single Raman peak at 302 cm**^−^**^1^ (gray dashed dotted line) corresponding to the transverse-optical (TO) phonon mode. The silicon-germanium sample exhibits three Raman peaks (blue solid line) associated with the optical vibrations of the Ge-Ge, Si-Ge, and Si-Si bonds in the alloy. Comparison of relative peak positions to literature^21^ suggests an x=0.75 alloy, i.e., Si_0.25_Ge_0.75_, in the experiment (see the text for details).

Recent developments in transient absorption (TA) spectroscopy in the extreme ultraviolet (XUV) provide new capabilities for femtosecond to sub-femtosecond time resolution and for direct access to electronic structural features.^5^ XUV ultrafast solid-state spectroscopy has opened up the possibility to study the dielectric response of insulators^6,7^ and electron dynamics in semiconductors.^8–12^ Recently, it was demonstrated that this technique can be employed for tracking electrons and holes as well as the energy shift of bands simultaneously at the M_4,5_-edge of germanium.^13^ In XUV transient absorption spectroscopy, a visible-to-near infrared (VIS-NIR) pump pulse that photoexcites carriers is followed after a time delay τ by a broadband XUV probe pulse generated by high harmonic generation (HHG)^14^ [Fig. 1(b)]. The XUV pulse excites core-level electrons into the valence band (VB) and conduction band (CB), and the transient absorption of the XUV photons tracks the dynamics of excited carriers and possible band modifications in the material. The element specificity of core-level XUV absorption renders this technique advantageous for site-specific investigations in heteroatomic, ternary, and quaternary systems.

The heteroatomic system of silicon-germanium alloy offers a wide range of applications, due to increased degrees of freedom in material design over monatomic semiconductors.^15^ This alloy has gained interest as it provides a higher mobility in strained semiconductor layers and thus offers the capability for higher frequency switching.^16^ Another major advantage for industrial applications is that it can be processed using standard silicon technology and can be grown on silicon, allowing for highly integrated devices. The silicon-germanium alloy has a tunable band gap and is highly useful in developing photovoltaics^17^ and high-speed electronics.^18^ Silicon-germanium alloys Si_1-x_Ge_x_ can be synthesized in any molar ratio of silicon and germanium, where x is the molar mass fraction. For x>0.85, the alloy becomes Ge-like where the direct band gap exhibits similar energy compared to the indirect gap at the L point. In contrast, for x<0.85, the alloy has Si-like properties with an indirect band gap at the X point whereas the direct optical gap is much larger than the indirect gap.^19^ The designation of Si-like and Ge-like alloy is based on the band structure (BS) and more specifically the nature of the band gap rather than on the fraction of atoms in the alloy.

Here, we employ ultrafast XUV transient absorption spectroscopy to investigate the carrier dynamics of silicon-germanium (Si_0.25_Ge_0.75_) alloy. An XUV continuum that spans a spectral range of more than 20 eV is generated to trace the carrier distributions in the alloy. The samples are nanocrystalline with domain sizes of ∼2.5 nm, grown by low-pressure chemical vapor deposition (LPCVD). Herein, the germanium atoms are employed as reporter atoms^20^ for probing the carrier dynamics in the alloy with sensitivity to electrons and holes. The transient absorption (TA) data is captured at the M_4,5_-edge of the germanium atoms in the silicon-germanium alloy and, by decomposing the TA data into contributions of electronic state blocking of optically excited carriers and band shifts, the carrier dynamics are retrieved (see also supplementary material, Sec. S1). The observations in the alloy are compared to recent results for nanocrystalline germanium.^13^ Notably, a midgap state is observed in the SiGe alloy that does not occur in germanium itself.

## II. METHODS

The experimental layout is depicted in Fig. 1(a). A VIS-NIR carrier-envelope-phase-stabilized (CEP) pulse with ∼4 fs pulse duration is used to generate a broadband XUV pulse in a gas cell filled with 28 Torr of xenon. Polarization-assisted amplitude gating (PASSAGE)^22^ allows for optimizing the XUV continuum [see pulse characterization in supplementary material, Sec. S3, the ∼4 fs pulse spectrum and phase that generate HHG is shown in Fig. S3(a) and the corresponding time domain in Fig. S3(c)]. A small fraction of the VIS-NIR pulse is split off as a pump pulse, time delayed, and focused collinearly with the XUV probe pulse onto the sample. The pump pulse duration is measured to 5.4 fs [spectrum with spectral phase and time domain characterization is shown in Figs. S3(b) and S3(c), respectively]. An aluminum filter removes the pump light, and the transmitted XUV light is spectrally analyzed by a flat-field spectrometer comprising a grating and X-ray CCD camera. A shutter periodically blocks the pump arm in order to acquire transient absorption spectra ${\Delta A}_{\text{meas}}\left(E,\tau \right)=A_{p}\left(E,\tau \right)-A\left(E\right)$, which are defined as the difference between the absorbance of the excited (pump on) absorbance $A_{p}$ and the static absorbance A (pump off) for different time delays τ. The experiment is performed at a repetition rate of 100 Hz in order to reduce heating of the sample. The integration time per spectrum was set to 1 s, i.e., one hundred laser pulses, in order to exploit the full dynamic range of the detector. The thin-film samples are raster-scanned on a grid spaced by 150 *μ*m over an area of ∼2.5 × 2.5 mm^2^. The positions within the grid are randomized over the time delay scan. Pump-on and pump-off spectra are separately measured on each sample at each time delay τ. In addition to the Si-like silicon-germanium thin film and the nanocrystalline germanium thin film, both of which are 100 nm thick, a gas cell filled with argon is periodically moved into the beam. A full sequence, i.e., taking six spectra (pump on/off on three samples) and translating samples into the beam, takes about 8 s. Measurements of germanium and silicon-germanium alloy were performed at the same time for consistency in comparing the ultrafast photoresponse of the two materials. At each time delay, XUV absorption spectra of germanium and silicon-germanium alloy with and without 800 nm photoexcitation are taken in series and this procedure is subsequently performed for all time steps. The noise level on the raw experimental data for both samples is ∼ΔA = 5 × 10^−3^.

Line shape changes in the argon 3s3p^6^4p autoionizing state^23^ are used for *in situ* correction of time-delay drifts in the system and to calibrate time zero (see also supplementary material, Sec. S3). The VIS-NIR pump pulse has an energy of 2.9 *μ*J resulting in an intensity of ${∼2\times 10}^{11}\;W/{\text{cm}}^{2}$ inside the semiconductor samples (note for the refractive index $n_{\text{Ge}}\approx n_{{\text{Si}}_{0.25}{\text{Ge}}_{0.75}}\approx 4.5\pm 0.2$ around 800 nm, Refs. 24 and 25) generating carrier densities of $N_{e,\text{Ge}}≅8\times 10^{20}\frac{1}{\text{cm}³}$ and $N_{e,\text{SiGe}}≅5\times 10^{20}\frac{1}{\text{cm}³}$ in germanium and silicon-germanium, respectively. Transient absorption data of both germanium and silicon germanium thin films shown here consist of five averages and the time delay τ was scanned with 0.6 fs time steps around time zero and with 3.3 fs time steps out to 1.5 ps. An upper bound for the instrumental response function was determined to be ∼6 fs by transient absorption measurements in argon. An in-depth description of the apparatus and more technical details are reported elsewhere.^13^ A detailed characterization of the optical setup can be found in supplementary material, Sec. S3. A detailed report on XUV transient absorption measurements in germanium can be found elsewhere;^13^ here, we refer to a specific set of measurements that were performed at the same time on both materials measuring both at each time delay step, i.e., in a non-sequential manner.

The electronic band structure (BS) of Si_0.25_Ge_0.75_ and the pump-probe scheme are depicted in Fig. 1(b) schematically [(detailed band structure in supplementary material, Fig. S2(a)]. The band structure is obtained from density functional theory (DFT)^26^ pseudopotential calculations carried out within the Virtual Crystal Approximation (VCA)^27^ using the QUANTUMESPRESSO code.^28^ Accordingly, the Si_0.25_Ge_0.75_ random alloy assuming diamond crystal structure is described by a single effective pseudopotential built from a linear combination of norm-conserving Ge and Si pseudopotentials^29^ including only the outermost occupied *s* and *p* shells. A plane wave kinetic energy cutoff of 100 Ry is employed and Brillouin zone integration is carried out over an 8 × 8 × 8 *k*-point grid. Exchange-correlation effects are treated at the level of the local-density approximation (LDA).^30^ Since the band gaps of both Ge and Si are underestimated within DFT-LDA, a post-processing scissors correction of 0.441 eV is applied to the band gap of the alloy. This correction is calculated as a linear combination of the individual band gap corrections for pure Si and Ge weighted in proportion to their respective alloy fractions. The corrected indirect band gap of 0.99 eV obtained from the calculation for the Si_0.25_Ge_0.75_ alloy is in a reasonable agreement with the value of 0.96 eV predicted by the tight binding calculations of Niquet *et al.* (Ref. 31) as well as experimental findings reporting 0.95 eV (Refs. 19 and 32). Further, conduction band minima (CBM) at other critical points obtained from the band structure calculation [see supplementary material, Fig. S2(a), for details] are consistent with electroreflectance measurements.^32^

For the pump VIS-NIR pulse, which is centered around 1.65 eV photon energy (see the spectrum in supplementary material, Fig. S4), direct transitions into the Γ valley and indirect transitions into the X, L, and K valleys are possible. In Sec. III, we will show that carriers below the direct band gap are observed in the XUV transient absorption at zero time delay, indicating that a significant portion of carriers are photoexcited into the X and L valleys through phonon-mediated indirect transitions. This is in contrast to the germanium measurement where the photon energies of the pump pulse are completely above the optical gap (0.8 eV) such that the carriers are excited predominantly through direct transitions in to the Γ valley.^13^

**FIG. 2.**
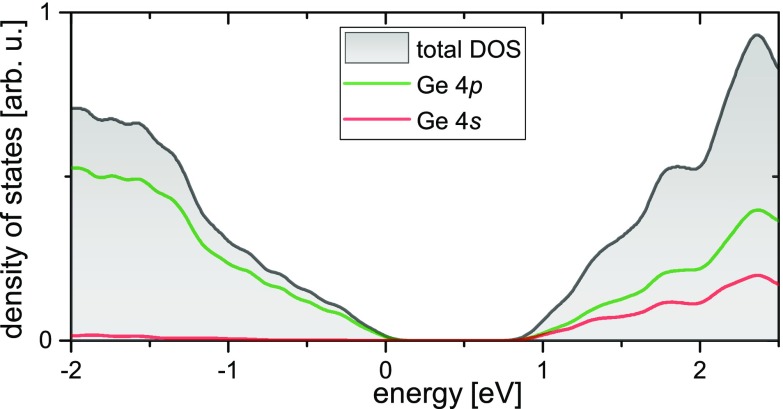
Density of states in Si_0.25_Ge_0.75_ calculated using density functional theory (DFT). Bands with 4*p* orbital character can be probed via transitions from the 3*d* core-levels. Similar to pure germanium (Ref. 13), the valence band is almost primarily of 4*p* orbital character, while the conduction band is a mix of 4*s* and 4*p* orbital character. Midgap features are not included in this calculation, since a crystalline super-cell containing 75% Ge atoms and 25% Si atoms was assumed and defects causing trap states were not considered.

Employing the Ge atoms as reporters for the transient state in the alloy, the broadband XUV pulse [violet arrows in Fig. 1(b)] probes the transient states in the VB and CB at the Ge M_4,5_-edge around 30 eV [Fig. 1(c)], which corresponds to excitation from the spin-orbit split 3*d*_3/2_ and 3*d*_5/2_ core electronic states. Transitions are possible to VB and CB states that are of 4*p* orbital character. First principles calculations using density functional theory (DFT) reveal that the VB is almost entirely of 4*p* orbital character and the CB is approximately 50% 4*p* character in the density of states (DOS) (Fig. 2). The total and partial DOS (PDOS) of Si_0.25_Ge_0.75_ alloy were calculated by a DFT simulation of an ordered 4 atom supercell containing 3 Ge and 1 Si atoms described by norm-conserving pseudopotentials. The supercell was constructed by doubling the fcc primitive cell of Ge along the first lattice vector direction and substituting one Ge atom with Si. In contrast to the VCA simulation employed earlier for the band-structure calculation, such supercell simulations allow for the Ge 4*p* and 4*s* partial DOS to be estimated independently of Si-derived PDOS contributions while neglecting the effects of random disorder. The latter nevertheless yields an overall density of states around the bandgap very similar to the VCA simulation that approximates random disorder [see supplementary material, Fig. 2(b)]. The QUANTUMESPRESSO code was used to simulate the PDOS employing the same numerical parameters as before. A Γ-centered 12 × 24 × 24 *k*-point grid was used for the DOS calculation shown in Fig. 2.

The 100-nm-thick silicon-germanium alloy sample was fabricated by LPCVD^33^ at 410 °C on a 30 nm thick silicon nitride membrane. For 20 min, a silicon nucleation layer (100 sccm Si_2_H_6_, 300 mTorr pressure) was deposited, followed by flowing 160 sccm of SiH_4_ and 40 sccm of GeH_4_ to deposit a silicon-germanium alloy. XRD analysis confirmed the nanocrystalline structure of the sample (see supplementary materials, Fig. S5). Measuring the Raman spectra of the silicon-germanium film [Fig. 1(d), solid blue line] reveals three characteristic Raman peaks corresponding to the optical vibrations of the Si-Si, Si-Ge and Ge-Ge bonds in the alloy. The molar fraction of germanium can be determined by analyzing the relative wavenumbers. Using the equations for the three peak wavenumbers given in Ref. 21, the spectrum suggests $x=0.75_{-0.03}^{+0.04}$ for the sample used here. Further, the Raman spectra confirm proper alloying in the LPCVD deposition, since there is no evidence for a Si or Ge transverse optical (TO) phonon mode at the expected values of 521 cm^−1^ and 302 cm^−1^, respectively. As expected, only the strong 302 cm^−1^ Ge TO phonon mode can be observed in the pure germanium sample [gray dashed-dotted line in Fig. 1(d)]. The determined molar mass fraction of x=0.75 suggests a Si-like silicon-germanium alloy,^19^ which is also qualitatively confirmed by the observed reduced optical absorption below 1.7 eV, i.e., less efficient indirect phonon-assisted excitation, in contrast to direct-gap germanium (supplementary material, Fig. S4). In addition, silicon-germanium alloy fabricated by LPCVD is known to exhibit a large number of point defects due to lattice size mismatch between silicon and germanium. Point defects result in dangling bonds and possible hydrogen contamination due to the fabrication process. However, the absence of distinct vibrational peaks from Si-H and Ge-H bonds^34^ in the Raman measurement [640 cm^−1^ and 565 cm^−1^, cf. Fig. 1(d)] indicates that the sample used has negligible hydrogen content. The presence of point defects in the sample also implies the presence of midgap states from localized defects and increased probability for carrier trapping.

After capturing the transient absorption spectra ${\Delta A}_{\text{meas}}\left(E,\tau \right)$, the data are processed as outlined in Ref. 13. Briefly, using a measured static absorbance [Fig. 1(c)], the measured transient absorption spectra can be decomposed into three components: state blocking, band shifts, and broadening (see supplementary material, Fig. S1). State blocking occurs when photoexcited electrons block otherwise possible XUV transitions in the CB, thus decreasing the absorption. Likewise, holes that are created in the VB by photoexcitation open up XUV transitions and increase the absorption. Band shifts can, for instance, be caused by band gap renormalization due to carrier-carrier screening in the VB and CB,^35^ by phonon renormalization, or by core-level shifts due to altered screening of the valence potential.^36,37^ These shifts of the excited state spectrum produce broad features in the transient absorption spectra, whose shape and magnitude mainly depend on the shape and steepness of the edge structure. Broadening of the excited state spectra^38^ can be induced by lifetime changes of the valence and/or core-level states following photoexcitation. The individual contributions can be retrieved from the transient absorption spectra by an iterative procedure outlined in-depth in Ref. 13. Furthermore, since the germanium M_4,5_-edge has contributions by two 3*d* core-level states that exhibit a spin-orbit splitting (${\Delta E}_{\text{so}}=0.58\;\text{eV}$, Ref. 39) comparable to the band gap in the materials considered here (${\Delta E}_{\text{gap},\text{Ge},\text{direct}}=0.8\;\text{eV}$, ${\Delta E}_{\text{gap},{\text{Si}}_{0.25}{\text{Ge}}_{0.75}\text{indirect}}=0.95\;\text{eV}$), it is necessary to separate these contributions, which is done by a Fourier method.^13^ In the following, we restrict ourselves to describing the energy and time-dependent state blocking of the contribution due to the 3*d*_5/2_ core-level ${\Delta A}_{\text{SB},{3d}_{5/2}}\left(E,\tau \right)$, obtaining a direct representation of the dynamics of electrons and holes versus energy and time delay. Here referred to as *carrier dynamics*, in Si-like silicon-germanium alloy and monatomic germanium for comparison.

## III. RESULTS AND DISCUSSION

**FIG. 3.**
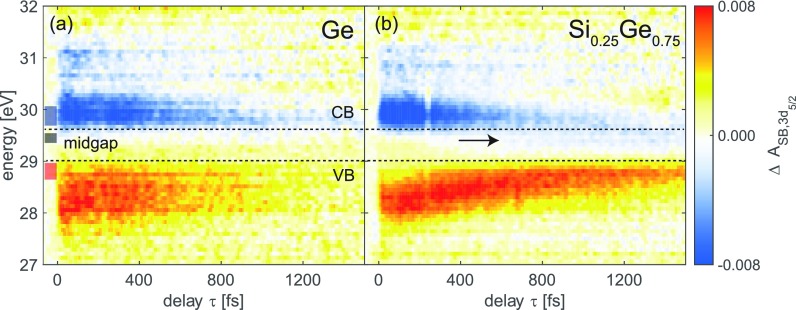
Time-resolved state blocking in germanium and silicon-germanium alloy measured in the XUV and spin-orbit separated, so that only the signal probed from the Ge 3*d_5/2_* core-level is shown. (a) Carrier dynamics in nanocrystalline germanium reveal the decays of the valence band (VB) and conduction band (CB) populations. Adapted from Ref. 13. (b) Direct comparison with silicon-germanium alloy shows that the Ge M_4,5_-edge can be employed to study carrier dynamics in the alloy. In the silicon-germanium alloy, an additional weak feature in the midgap (indicated by the black arrow), which is assigned to trap states, can be observed at longer time delays (detailed analysis in Fig. 5). The three colored rectangles on the left in (a) indicate the energies of the line profiles in Fig. 5.

Here, high harmonic XUV light is established as a probe of electron dynamics through core-level transitions in semiconductors as a function of time. In Fig. 3, the carrier dynamics of germanium^13^ and silicon-germanium versus time delay τ are depicted. A positive time delay represents the NIR-VIS pump pulse arriving first and the broadband XUV pulse arriving later to probe the transient states. According to Ref. 13, the CB and VB in germanium are mapped to energies greater than 29.6 eV and less than 28.9 eV in the XUV, respectively [Fig. 3(a)]. The general similarities between the transients of germanium and silicon-germanium [Fig. 3(b)] strongly corroborate that the reporter atom concept^20^ is successfully applied here to solids and that germanium atoms can be employed to probe the carrier dynamics of electrons and holes in the Si-like indirect gap alloy. Other recent findings by Santomauro *et al.*^40^ suggest that this is expected to be true for carriers that are either localized near the germanium atoms or delocalized in the alloy.

In nanocrystalline germanium [Fig. 3(a)], the carriers appear to decay symmetrically, which was described by a fast trap-assisted recombination^41–44^ in which the lifetimes of the carriers associated with the trap are short (time constant of ∼1.1 ps).^13^ In contrast, there is an asymmetry in silicon-germanium [Fig. 3(b)], i.e., the electrons decay faster than the holes, whereas the hot holes relax up towards the VB edge during the first ∼400 fs and only slowly decay thereafter. The second apparent difference is that a signal with negative sign (less absorption) starts growing in the midgap of the alloy after ∼400 fs, continuously increasing towards the largest measured time delay of 1.5 ps [indicated by the black arrow in Fig. 3(b)].

**FIG. 4.**
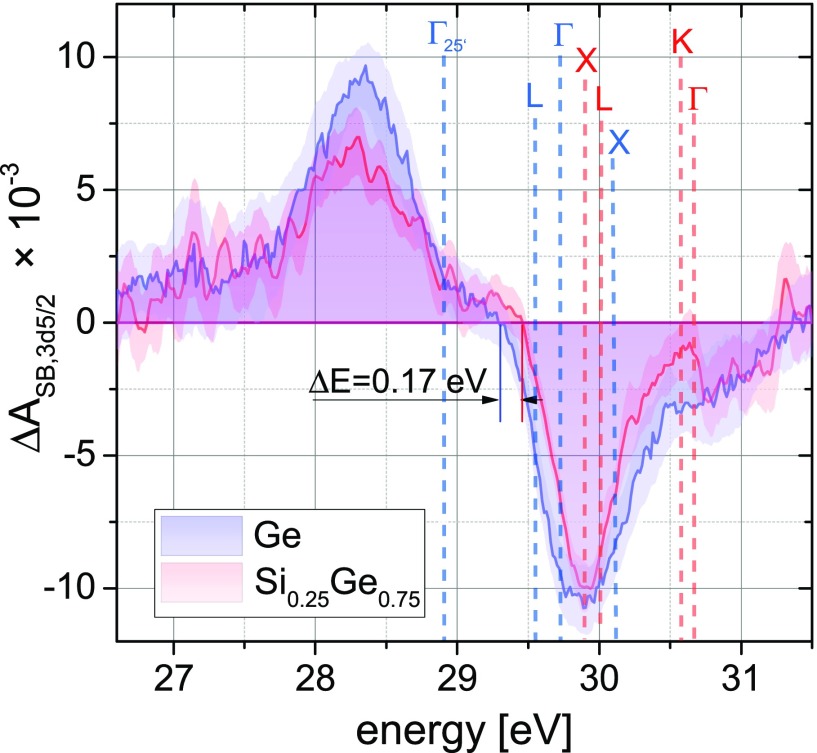
Comparison of the state blocking following photoexcitation after ∼10 fs in germanium and silicon-germanium alloy. A time slice of the state blocking averaged from τ = 8,…,12 fs is shown, which precedes the onset of carrier relaxation. Although the valence band (VB) distribution features a similar shape, the conduction band (CB) onset in silicon-germanium is shifted to higher energies indicating a larger band gap. At the zero-crossing, an increase of the band gap by 0.17 eV compared to germanium can be measured. The letters designate valley assignments for germanium (blue letters) and silicon-germanium (red letters, cf. Fig. S5). The letters L, Γ, X, and K designate the conduction band minima in the respective valleys calculated for x=0.75 in relation to the valence band maximum at Γ_25'_.

It is instructive to compare the initial carrier distributions in the two materials, i.e., before relaxation processes set in. In Fig. 4, an energy slice of the data in Fig. 3 averaged from +8 to 12 fs is shown for both materials, i.e., immediately following photoexcitation. With the valence band maximum (Γ_25'_ critical point) at approximately 28.9 eV (Ref. 13), the conduction band minimum at the Γ point of the silicon-germanium alloy appears at 30.6 eV, indicating that carriers excited over the direct gap should appear at energies above 30.6 eV. However, the initial carrier distribution in the conduction band spans from 31.5 eV down to about 29.6 eV, and the largest absolute value of the transient absorption signal in the CB appears at approximately 30 eV (Fig. 4). This implies that a significant portion of carriers is directly excited into the X and L valley by an indirect phonon-assisted process. For energies between 27 eV to 29 eV, corresponding to the VB, both systems have comparable spectral distributions. On the CB side, the onset of electron features is blue-shifted by ΔE=0.17 eV in silicon-germanium compared to germanium (Fig. 4) due to the band energies of silicon and germanium, which increases the band gap compared to pure germanium. For an alloy with x=0.75, the indirect band gap is given by the energy difference between the X valley in the CB and the Γ valley in the VB^19^ [cf. Fig. 1(b)]. The indirect band gap ${\Delta E}_{\Gamma -X}\;$(excitation from Γ point VB valley to the CB X valley) for x=0.75 is 0.95 eV (Refs. 19 and 32). With the direct band gap in germanium being ${\Delta E}_{\text{Gap},\text{Ge}}=0.8\;\text{eV}$, the measured blue shift of the CB can be explained by the band alignment in the alloy. Note that the XUV transient absorption measurements show that the increased band gap is solely due to blue-shifting of the CB edge, whereas the energy of the valence band remains as in pure germanium in relation to the Ge 3*d* core-levels within the instrumental resolution accessible here (${\delta E}_{\text{inst}}\approx 0.07\;\text{eV}$).

Comparing the critical points or valleys in the CB (Fig. 4, letters in blue for germanium and red for silicon-germanium), one finds that in the Si-like alloy the X critical point is redshifted, whereas the L and Γ critical points are blueshifted compared to germanium. The amount of blueshift of the Γ point is approximately 0.9 eV for x=0.75, which aligns the Γ critical point closely with the K critical point [cf. supplementary material, Fig. S2(a)]. Since for the same photon energy excitations into the K valley constitute an indirect transition, it is expected that the majority of the excitation that reaches the Γ/K critical points will excite into the Γ valley. The L and X critical points are energetically too close to be separated in the experiment, given that the core-hole life time $\tau_{3d}$ of the Ge atoms limits the achievable energy resolution to ${\delta E}_{\text{Ge},\;\tau_{3d}}\approx 0.24\;\text{eV}$ (Ref. 39). In the silicon-germanium experiment (red shaded area in Fig. 4), the main excitation in the initial carrier distribution aligns with the X and L valleys, but a second weaker maximum can be observed at about ∼0.8 eV higher photon energy, signifying excitations of carriers excited to the Γ valley. Although XUV transient absorption on randomly oriented crystallites with linearly polarized pump and probe in the same polarization state does not resolve electron and crystal momenta, the X/L versus Γ/K valleys appear as discernible features due to the increased densities of states at the energies corresponding to these valleys.

**FIG. 5.**
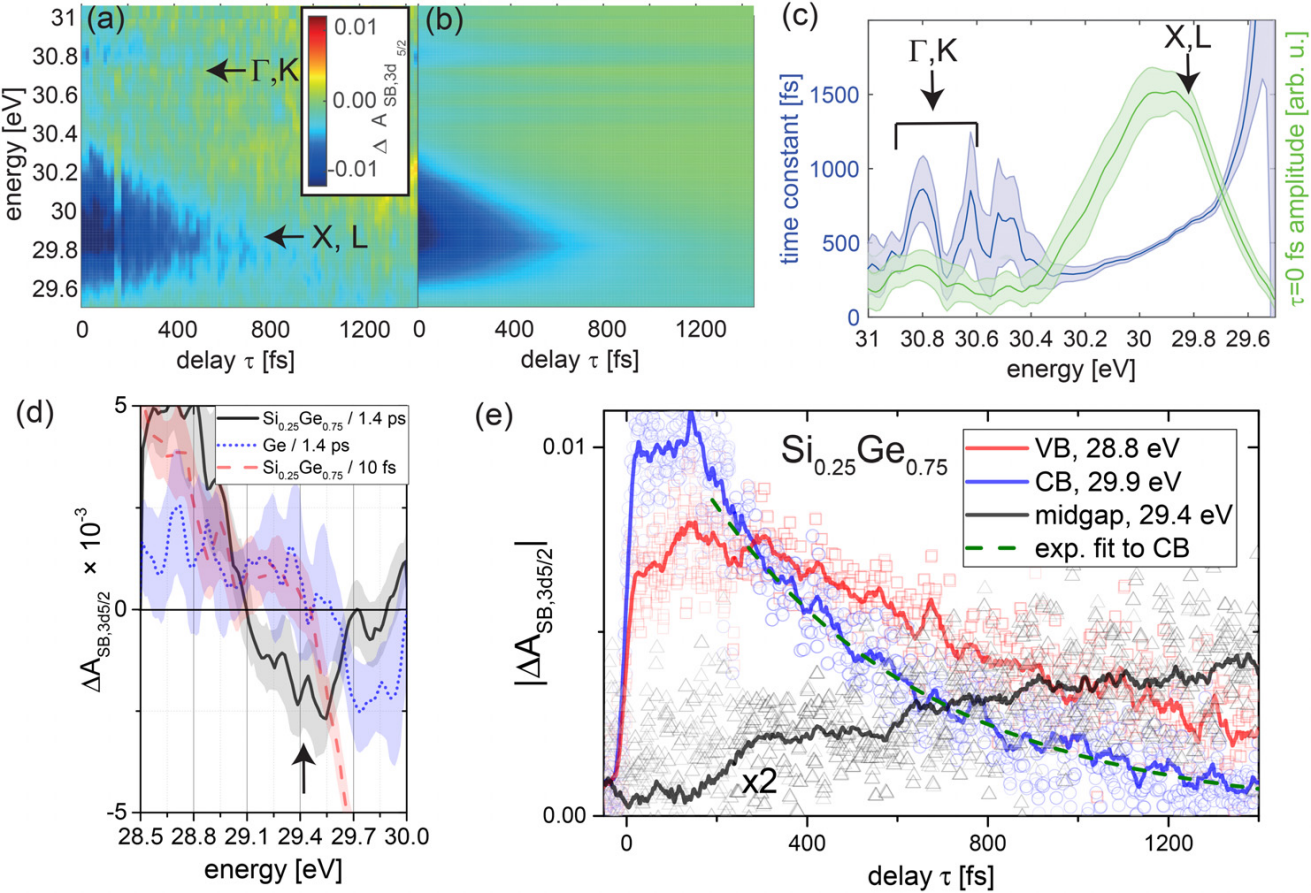
Kinetic analysis of electrons, holes, and midgap feature. Panels (a) to (c) analyze the conduction band (CB) kinetics in silicon-germanium. (a) At each energy, a single exponential is fit to the experimental data. (b) Using the retrieved initial amplitudes and time constants as a function of energy, the dynamics of the transient signal are captured. (c) The initial amplitude of the fit (green line with shaded error bar) depicts the initial electron distribution over energy. The two maxima correspond to an indirect excitation to the X and L as well as the direct excitation into the Γ bands. The time constant (blue line with a shaded error bar) over energy indicate the average life times of the carriers. (d) After hot carrier relaxation (τ = 1.4 ps), a weak negative feature (indicated by the black arrow) becomes visible in the midgap region of the alloy (black line with shaded error bars). For comparison, in germanium, no significant signal is observed (blue dotted line) at these time delays. Initially the midgap region between 29.1 eV and 29.4 eV in silicon-germanium (red dashed line) exhibits a near-zero signal indicative of the absence of carrier population in the band gap region. (e) In silicon-germanium alloy, a faster decay of the electrons (time constant ∼0.9 ps) and slower decay for the holes are observed. At the same time, a midgap feature grows in. Note that here the absolute value of the signals is plotted for better comparison of the temporal behavior. The energy ranges for extracting the line profiles are indicated by the three rectangles in respective colors in Fig. 3(a). The signal of the midgap feature has been amplified by a factor of two to increase visibility.

In order to analyze the electron kinetics in the CB of silicon-germanium [Fig. 5(a)], single exponentials are fit to each slice along the time delay axis with the initial amplitude at τ=0 fs and a decay constant. Representing the fit result as a 2D map of energy versus time delay [Fig. 5(b)] shows good agreement with the data. The main feature around 29.9 eV has been assigned to the X and L valleys (cf. Fig. 4). The initial amplitudes [green line with shaded error bar in Fig. 5(c)] resemble the initial electron distribution, which compares to the CB signal in Fig. 4. It becomes apparent that the center of mass of the initial amplitude distribution lies above the CB valleys, indicating hot electrons with excess energy. At the same time, the short lifetimes of less than 400 fs (blue line with shaded error bars) at energies higher in the X/L bands between 30 and 30.2 eV indicate a fast relaxation of these hot electrons towards the X and L valley, where the lifetime continually increases, indicating that the carriers accumulate in the valleys. The process of hot electron relaxation towards a valley, i.e., intravalley scattering, is mainly mediated by carriers scattering with acoustic phonons, although optical phonons can be involved depending on the excited valley. Carriers in the L valley will ultimately scatter to the X valley and accumulate in the lower lying X valley. This relaxation pathway is more likely due to the electrons reducing their energy further by scattering into the X valley. For a recombination with holes from the VB, a symmetric decay of carrier signals would be expected [compare to germanium in Fig. 3(a)]. The decay, i.e., carrier removal, that takes place on a time scale of ∼900 fs in the X valley [see the CB edge region around 29.7 eV in Figs. 5(a)–5(c)], can be understood as either CB electrons scattering into trap states or electrons scattering into the VB by carrier recombination. Both processes can be mediated by carrier-phonon scattering, carrier-carrier scattering, or Auger processes.^45^ At around 30.8 eV, a weaker feature with a less pronounced but similar shape is observed and attributed to electrons initially excited predominantly near the Γ point. Here, a comparable lifetime on the order of 0.9 ps is measured, which suggests that the phonon-assisted scattering from the Γ valley into the X or L valley, to further relax the electrons, is inefficient compared to what is observed in the germanium nanocrystal samples, where the lifetimes of higher lying CB valleys were significantly shorter than the lifetimes in the lower lying valleys.^13^ These differences, however, may be due to the crystalline morphology and numbers of trap states in the two different samples. The relatively weak and sharp features at 30.6 and 30.5 eV [see Figs. 5(a) and 5(c)] could potentially relate to a weak excitation into the K valley, but the energetic proximity to the Γ critical point within the spectral resolution given by the natural lifetime of the Ge 3*d* core-level and the signal-to-noise ratio prohibit detailed analysis.

Figure 5(d) shows energy slices for germanium and the Si_0.25_Ge_0.75_ alloy at a longer time delay (τ = 1.4 ps). The weak midgap feature becomes clearly visible in the alloy at energies between 29.1 and 29.5 eV (black line). In germanium, the signal is zero within the error bars (dotted blue line), which can also be qualitatively assessed in Fig. 3(a). Comparing to the early signal (dashed red line) after excitation, one finds a shallow signal around zero absorbance in the respective midgap region (29.1–29.5 eV), as is expected for a band gap. This suggests that the midgap feature is due to trap states tentatively above the Fermi energy, which are not directly accessible by the VIS-NIR laser. These states can be populated after hot electrons relax to the CB edge from where the electrons subsequently scatter into these states. X-ray photoelectron spectroscopy studies on silicon-germanium alloys suggested the existence of these states mainly being localized on the germanium atoms and exhibiting *p* orbital character,^46^ which together corroborates observability in the XUV transient absorption experiment.

The electron dynamics can be further assessed in the time profiles taken near the CB and VB edge as well as the midgap region [Fig. 5(e); see also Fig. 2(a) for shaded rectangles indicating the energy range used for integrating the signals shown here]. The qualitatively observed trend in silicon-germanium showing asymmetric signal decay becomes visible in the VB and CB line profiles in Fig. 5(e). The signal associated with the electrons at the CB edge (X/L valleys) decays with a time constant of ∼0.9 ps in silicon-germanium and follows a single exponential decay [the green dashed line in Fig. 5(e)] as discussed in detail in the previous paragraphs. At the same time, although the CB signal decays, a signal grows in the midgap [black line in Fig. 5(e)]. The midgap feature is found to have a negative sign [cf. Fig. 5(d)], which is indicative of electrons contributing to the state blocking. Qualitatively the sign and relative energy of the midgap feature corroborates the assignment of the midgap states to trap states near the CB edge^47^ into which the electrons relax and accumulate. This observation suggests that electrons scatter from the bottom of the CB into nearby trap states where a significant population of electrons builds up, so that population can be observed in the XUV. This buildup further suggests that carrier recombination with holes takes place on a time scale longer than measured here. This is supported by the hot holes in the VB relaxing towards the VB edge during the first ∼400 fs following photoexcitation [see the blueshift of the VB feature in Fig. 2(b)], but in general only exhibiting a slower decaying signal [red line in Fig. 5(b)] compared to the electrons at the CB edge. Combined, this supports a picture where after ∼1.5 ps, a large number of electrons is trapped in states near the CB edge at the X point, whereas the holes accumulate at the VB edge near the Γ point. The carrier recombination across the indirect band gap subsequently requires carrier-phonon scattering to overcome the momentum difference, which in principle would render the recombination process, which is similar to the phonon-assisted excitation process, less efficient compared to materials that have a direct band gap.

## IV. CONCLUSION

In this work, XUV transient absorption measurements allow for probing of electron dynamics in a silicon-germanium alloy. The ability to resolve lifetimes of different valleys in the CB after excitation across the direct and indirect band gap simultaneously with hole dynamics adds to capabilities that optical pump- XUV probe techniques offer. In the present experiments, the germanium atoms serve as reporter atoms for the alloy. This method, employing XUV absorption from core-levels, allows direct access to the dynamics of both carrier species that are relevant for the electronic properties of a semiconductor with ultrafast temporal resolution. Silicon-germanium with a germanium content of 75% as used in this work is an indirect band gap material and the CB has silicon-like properties. It is found that the CB in the alloy exhibits a static blueshift compared to monatomic germanium thus effectively increasing the band gap, while the alignment of the VB with respect to the 3*d* core-level is the same as in pure germanium. The large spacing of the X/L versus Γ CB valleys in the alloy enables valley-sensitive lifetime measurements between carriers excited across the direct versus indirect gap. The comparable lifetime of carriers in the X/L versus Γ valleys suggests a small scattering cross section for the higher lying Γ valley towards the X valley, in contrast to germanium where previously it was observed that decreasing lifetimes occur for increased energy above the lowest band edge. A specific feature observed in the alloy is a midgap feature that is assigned to trap states near the CB minimum into which electrons accumulate following intravalley relaxation. The indirect gap in the silicon-like alloy appears to reduce the cross section for recombination with the holes that accumulate at the top of the VB, causing an asymmetry in the carrier decay between VB and CB. A more quantitative analysis would require knowledge of the *k*-dependent transition dipoles for the XUV and knowledge about density and localization of these trap states.

A next step for employing XUV transient absorption spectroscopy for site-specific study of silicon-germanium alloys can involve measuring the dynamics at the silicon L_2,3_-edge which allows access to other CB and possibly midgap features in addition to the germanium M_4,5_-edge features observed here. This could reveal further insight into the localization and kinetics of the trap states. The direct and valley-resolved access into the VB and CB with, in principle, sub-femtosecond temporal resolution renders germanium an ideal component in alloys for studying carrier dynamics using XUV transient absorption spectroscopy. Further, the measurements can be employed along with first principles calculations to improve the understanding of photoexcitation in indirect band gap materials.^48^ The findings presented here hold great promise for studying carrier dynamics in ternary and quaternary semiconductor alloys that include fractions of germanium, which are becoming increasingly important for highly integrated and highly efficient photonics devices.^16,49,50^

## V. SUPPLEMENTARY MATERIAL

See supplementary material for the result of decomposing the transient absorption data into components of electronic state blocking, XUV absorption edge shifts and broadening; a detailed electronic band structure; a characterization for both 800 nm pulses for high harmonic generation and photoexcitation, and a characterization of the samples.
